# Postpartum Emergency Department Use Following Midwifery-Model vs Obstetrics-Model Care

**DOI:** 10.1001/jamanetworkopen.2024.8676

**Published:** 2024-04-29

**Authors:** Carla Sorbara, Joel G. Ray, Elizabeth K. Darling, Hannah Chung, Sho Podolsky, Therese A. Stukel

**Affiliations:** 1 Department of Obstetrics and Gynecology, North York General Hospital, Toronto, Ontario, Canada; 2 ICES, Toronto, Ontario, Canada; 3 Department of Obstetrics and Gynecology, St Michael’s Hospital, Toronto, Ontario, Canada; 4 Department of Obstetrics and Gynecology, McMaster University, Hamilton, Ontario, Canada; 5 Institute of Health Policy, Management, and Evaluation, University of Toronto, Toronto, Ontario, Canada

## Abstract

**Importance:**

Emergency department (ED) use postpartum is a common and often-preventable event. Unlike traditional obstetrics models, the Ontario midwifery model offers early care postpartum.

**Objective:**

To assess whether postpartum ED use differs between women who received perinatal care in midwifery-model care vs in traditional obstetrics-model care.

**Design, Setting, and Participants:**

This retrospective population-based cohort study took place in Ontario, Canada, where public health care is universally funded. Participants included women who were low risk and primiparous and gave birth to a live baby in an Ontario hospital between 2012 and 2018. Data were collected from April 2012 to March 2018 and analyzed from June 2022 to April 2023.

**Exposures:**

Perinatal care clinician, namely, a midwife or obstetrician.

**Main Outcome and Measures:**

**:** Any unscheduled ED visit 42 days postpartum or less. Poisson regression models compared ED use between women with midwifery-model care vs obstetrics-model care, weighting by propensity score-based overlap weights.

**Results:**

Among 104 995 primiparous women aged 11 to 50 years, those in midwifery-model care received a median (IQR) of 7 (6-8) postpartum visits, compared with 0 (0-1) visits among those receiving obstetrics-model care. Unscheduled ED visits 42 days or less postpartum occurred for 1549 of 23 124 women (6.7%) with midwifery-model care compared with 6902 of 81 871 women (8.4%) with traditional obstetrics-model care (adjusted relative risks [aRR], 0.78; 95% CI, 0.73-0.83). Similar aRRs were seen in women with a spontaneous vaginal birth (aRR, 0.71; 95% CI, 0.65-0.78) or assisted vaginal birth (aRR, 0.70; 95% CI, 0.59-0.82) but not those with a cesarean birth (aRR, 0.94; 95% CI, 0.86-1.03) or those with intrapartum transfer of care between a midwife and obstetrician (aRR, 0.94; 95% CI, 0.87-1.04). ED use 7 days or less postpartum was also lower among women receiving midwifery model care (aRR, 0.70; 95% CI, 0.65-0.77).

**Conclusions and Relevance:**

In this cohort study, midwifery-model care was associated with less postpartum ED use than traditional obstetrics-model care among women who had low risk and were primiparous, which may be due to early access to postpartum care provided by Ontario midwives.

## Key Points

**Question:**

Is care by a midwife compared with an obstetrician associated with different rates of emergency department (ED) use in the postpartum period?

**Findings:**

In this cohort study of 104 995 patients who were low risk, women receiving innovative midwifery-model care experienced a 22% lower risk of ED use post partum than patients receiving traditional obstetrics-model care.

**Meaning:**

These findings suggest that midwifery-model care, which offers early postpartum visits, may reduce maternal ED use after birth; this approach may fill an important gap in postpartum care.

## Teaser

This cohort study examines whether postpartum emergency department use differs among women who received perinatal care in a midwifery model vs in a traditional obstetrics model among women in Ontario, Canada.

## Introduction

In 2018, the Ontario Provincial Council for Maternal and Child Health highlighted several challenges with existing postpartum services in Ontario, Canada, including a lack of coordination and provision of care.^1^ Research about service use showed that approximately 5% of women who give birth have at least 1 emergency department (ED) visit in the first 10 days postpartum, possibly reflective of an unmet need for community-based postpartum services.^2,3,4^ A 2018 US study showed that approximately 75% of postpartum women who presented to the ED were of low acuity, with less than 25% being admitted to hospital.^5^ A Canadian study found that almost 40% of all women in the peripregnancy period have at least 1 ED visit, peaking in the first 5 days postpartum, when they are unlikely to have ready access to a maternity care clinician.^6^

In 1993, the Ontario Ministry of Health created the Ontario Midwifery Program, a provincially funded primary care maternal-newborn program that prioritizes 24-hour on-call service, postpartum home visits, and continuity of care.^10^ Midwives work alongside obstetrics and family medicine teams, collaborating with specialists when care becomes more complex.^7^ Midwives and obstetricians in Ontario work in clinician-specific care models.^8,9^ Midwives work in group practices, with continuity and on-call service throughout the antenatal, intrapartum, and postpartum periods. In the first week after birth, patients receiving midwifery care receive several routine clinical visits at home or in the midwife’s office, typically from the same midwife whom they met in the antenatal period. This is followed by several visits in the 6-week postpartum period comprising well-woman and well-baby checks, breastfeeding support, wound care, and monitoring or testing for ongoing maternal or newborn concerns.^11,8^ For urgent concerns, women receiving midwifery-model care have access to their midwives 24 hours a day via pager.^12^ In contrast, women receiving obstetrics-model care are typically discharged from hospital within 24 to 48 hours after birth, with limited formal coordination in transitioning between hospital and community care.^1^ Most obstetricians provide clinic-based antenatal care, with limited intrapartum continuity, no 24-hour on-call access for patients postpartum, and only a single routine follow-up visit at 6 weeks following birth^11^; postpartum care for a complication generally takes place through an emergency department (ED) or a family medicine office.^13^

The current study compared postpartum ED use between women who gave birth to a live baby in an Ontario hospital between 2012 to 2018 and received care in a midwifery-model or an obstetrics-model. It was hypothesized that perinatal midwifery-model care would be associated with a lower risk of postpartum ED use during the first 42 days postpartum.

## Methods

### Setting

This retrospective cohort study took place in Ontario, Canada. All women in the study were eligible for clinic and hospital pregnancy care funded by the universal Ontario Health Insurance Plan (OHIP). All in-patient, ED, and ambulatory visits were captured in health administrative databases.

### Ethics Approval

The data used in this study is authorized under section 45 of Ontario’s Personal Health Information Protection Act and does not require review by a research ethics board. This report follows Strengthening the Reporting of Observational Studies in Epidemiology (STROBE) reporting guideline reporting guidelines for observational studies.

### Study Population

We included all pregnant women who were primiparous, low risk, aged 11 to 50 years, and gave birth in an Ontario hospital between April 1, 2012, and February 1, 2018. We use the term woman, while acknowledging that not all those who give birth identify as female. A woman was deemed to be low risk if she had a singleton livebirth within hospital at 34 weeks gestation or more. Including only primiparous women eliminated the influence of a prior birth event (eg, preterm birth, cesarean delivery, or preeclampsia) on the choice of care clinician in the current pregnancy. We excluded women without a valid OHIP number, missing recorded body mass index (BMI; calculated as weight in kilograms divided by height in meters squared) or gestational age at birth, those who received family physician model care (ie, women who received all antenatal, intrapartum, and postpartum care from a family physician), and those with an unknown perinatal care clinician type. BMI was required since it is a risk factor for postpartum complications and was missing at random. Gestational age was necessary since birth at more than 34 weeks helped define the low-risk cohort. We excluded patients who used a family physician for their obstetric care because Ontario family physicians work in a variety of care models with varying degrees of continuity and on-call service,^9^ creating important heterogeneity relevant to our outcome that we could not measure. Additionally, unlike midwives and obstetricians, many family physicians provided primary care prior to pregnancy and after the postpartum period. We also excluded women with more than 2 ED visits within the 2-year period preceding the pregnancy because these patients tend to use the ED for routine primary care visits. We also excluded those discharged from midwifery-model care during the antenatal period, women whose hospital length of stay was more than 7 days following the birth (a likely reflection of a serious birth complication), and those who died during the birth hospitalization or up to 42 days thereafter.

A woman was considered to be exposed to midwifery-model care based on Better Outcomes and Registry Network (BORN) data elements designed specifically to capture patients receiving midwifery care who were billed in the capitation model as a midwifery patient.^14^ A woman was in the obstetrics-model group if she had an obstetrician as her admitting clinician and was not categorized as exposed to midwifery-model care.

### Data Sources

Existing deidentified patient records were linked using unique encoded identifiers and analyzed at ICES. ICES is an independent, nonprofit research institute whose legal status under Ontario’s health information privacy law allows it to collect and analyze health care and demographic data, without consent, for health system evaluation and improvement. Multiple linked Ontario health administrative databases were used containing information regarding all publicly insured hospital and care clinician services as follows: (1) the Discharge Abstract Database for hospital admissions, procedures, and transfers, including the most responsible diagnosis for length of stay, secondary diagnostic codes, comorbidities present upon admission, complications occurring during the hospital stay, and attending physician identifier; (2) the National Ambulatory Care Reporting System for ED visits; (3) the OHIP database for physician billings; (4) Refugees and Citizenship Canada Permanent Resident Database for immigrant status; (5) the Registered Persons Database (RPDB) for patient demographic information and deaths, and (6) Ontario Marginalization Index for neighborhood material resources.^28^ Midwifery and obstetric patient characteristics, including risk factors (eg, smoking and alcohol exposure during pregnancy), comorbidities, care indicators (eg, prenatal class attendance), complications, and delivery characteristics (eg, procedures received), were captured in the BORN information system (BIS). BIS is a prescribed maternal, newborn, and child registry under Ontario’s Personal Health Act, which collects, interprets, shares and protects high-quality data, and captures 100% of the births that take place in the province.^14,15^

### Study Outcomes

The primary outcome was any unscheduled ED visit within 42 days postpartum, starting from the date of discharge from hospital for the birth. This 42-day period captures complications associated with the birth, as well as persistent clinical issues that may arise in the standard 6-week postpartum period.^5^ Secondary outcomes included any ED visit within 7 days following the index hospital discharge date, admission to hospital within 42 days after hospital discharge, and any low-acuity ED visit within 42 days.

Analyses were further stratified by (1) mode of birth (spontaneous vaginal, assisted vaginal or cesarean section) and (2) by intrapartum transfer of care from a midwife to an obstetrician. While patients who have a transfer of care experience more complications during their labor and birth, the midwife remains with the patient for the delivery in almost every circumstance and also provides care following hospital discharge.^12^

### Statistical Analysis

We reported descriptive statistics for baseline variables and care indicators and used standardized differences to assess balance across exposure groups, with a difference of less than 0.1 suggesting adequate balance. Propensity score (PS)–based overlap weights were used to create cohorts that were balanced across baseline characteristics. The PS model was created using multivariable logistic regression, to estimate the likelihood of receipt of midwifery vs obstetrics-model care. The logistic regression model included covariates selected a priori to be associated with choice of care clinician type, including age (less than 19, 20 to 24, 25 to 29, 30 to 34, 35 to 39, and 40 years or older), BMI (underweight, normal weight, overweight, obese), immigration status (Canadian-born or not), primary language (English, other, unknown), material resources quintile (1 [least resources] to 5 [most resources], unknown), rurality (urban, rural, remote, unknown), smoking in pregnancy (none, any smoking, unknown), cohabitation with a person who smoked (no, yes, unknown), substance or alcohol abuse in pregnancy (none, any, unknown), and intimate partner violence (none, any, unknown). Also included were preexisting maternal health factors: physical (none, any [eg, essential hypertension or preexisting diabetes], unknown), mental (none, any, unknown), fertility assistance (none, any, unknown), and number of ED visits within 2 years before conception (0 or 1). While the BORN database is a rich data source, it relies on both the clinician capturing a patient’s details, and the patient disclosing that information. For baseline covariates and care indicators with missing or unknown values, we added a missing or unknown category.^14^

Each woman was then weighted according to an overlap weight (OW), defined as the probability of being assigned to the opposite exposure group based on the PS.^16,17^ OWs give larger weight to individuals who have a higher probability of receiving either exposure, being those with the greatest overlap in observed covariates and the most in equipoise. OWs downweight individuals in the extremes of the distribution, achieve perfect balance for all covariates included in the PS, and produce the smallest standard errors among all balancing weight approaches. Poisson regression models, weighted by OWs, estimated the adjusted relative risk (aRR) and 95% CI for any unscheduled ED visit among women with midwifery-model care vs obstetrics-model care, and included a robust variance estimator to account for the weighting. Adjusted absolute risk differences (ARD) were calculated using a modified logistic regression model based on marginal probabilities, and 95% CI estimated by bootstrapping with resampling between 200 and 1000 times.^18^

Statistical significance was set at *P* < .05 and tests were 2-sided. Statistical analysis was performed from June 2022 (cohort creation) to April 2023 (most recent outputs). All analyses were conducted using SAS version 9.4 (SAS Institute)

## Results

Of 684 011 valid birth records, 579 016 patients were excluded, including 391 631 (57%) who were not primiparous, 42 475 (6%) with a missing or implausible BMI, 11 856 (2%) who did not fit the low-risk criteria, 70 388 (10%) who received family physician-model care and 45 322 (7%) with 2 or more ED visits in the previous 2 years. Of the 104 995 women included in the study, 81 871 (78%) were in the obstetric-model group and 23 124 (22%) in the midwifery-model group (Figure). Women in obstetrics-model care were more likely to be older than women in the midwifery-model group (40 years or older, 2945 [3.6%] vs 376 [1.6%]), to live with obesity (12 624 [15.4%] vs 3134 [13.6%]), to live in an urban area (69 108 [84.4%] vs 17 594 [76.1%]), and to have immigrated to Canada (57 664 [70.4%] vs 20 566 [88.9%] Canadian-born) (Table 1). Women in obstetrics-model care were also more likely to use assisted reproduction than women in the midwifery-model group (5501 [6.7%] vs 1066 [4.6%]) and have 1 ED visit in the prior 2 years (24 849 [30.4%] vs 6 398 [27.7%]). Women in the obstetrics-model group were less likely to have English as their primary language than women in the midwifery-model group (63 052 [77.0%] vs 21 071 [91.1%]), to report alcohol use (1819 [2.2%] vs 565 [2.4%]) or substance use (1151 [1.4%] vs 223 [1.0%]), but were more likely to report smoking (5398 [6.6%] vs 856 [3.7%]) and cohabitation with an individual who smoked (10 958 [13.4%] vs 2128 [9.2%]).

**Figure.**
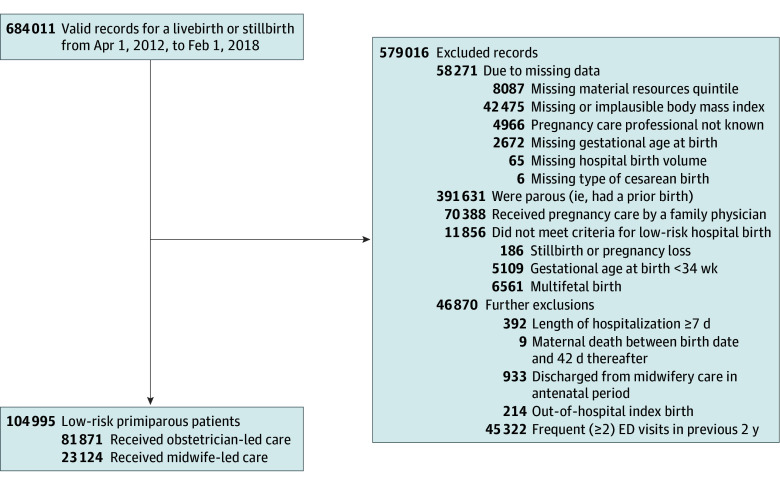
Flow Diagram ED indicates emergency department.

**Table 1.** Patient Characteristics According to Receipt of Midwifery-Model Care or Obstetrics-Model Care, Before and After Propensity Score Overlap Weighting

Characteristic	Before weighting			After weighting^a^		
	Patients, No. (%)		Standardized difference	Patients, %		Standardized difference
	Midwifery model (n = 23 124)	Obstetrics model (n = 81 871)		Midwifery model	Obstetrics model	
Maternal age, y						
<19	272 (1.2)	2121 (2.6)	0.10	1.5	1.5	0
20-24	1921 (8.3)	8802 (10.8)	0.08	8.9	8.9	0
25-29	7715 (33.4)	24 933 (30.5)	0.06	32.5	32.5	0
30-34	9801 (42.4)	30 909 (37.8)	0.10	41.3	41.3	0
35-39	3039 (13.1)	12 161 (14.9)	0.05	13.8	13.8	0
≥40	376 (1.6)	2945 (3.6)	0.12	2.0	2.0	0
Maternal BMI						
Underweight	918 (4.0)	4948 (6.0)	0.10	4.4	4.4	0
Normal weight	13 771 (59.6)	46 457 (56.7)	0.06	58.3	58.3	0
Overweight	5301 (22.9)	17 842 (21.8)	0.03	22.9	22.9	0
Obese	3134 (13.6)	12 624 (15.4)	0.05	14.5	14.5	0
Canadian born	20 566 (88.9)	57 664 (70.4)	0.47	85.7	85.7	0
Rurality index						
Urban	17 594 (76.1)	69 108 (84.4)	0.21	80.0	80.0	0
Nonurban	4355 (18.8)	10 839 (13.2)	0.15	17.4	17.4	0
Remote	1102 (4.8)	1786 (2.2)	0.14	3.5	3.5	0
Unknown	73 (0.3)	138 (0.2)	0.03	0.2	0.2	0
Prior ED visits						
0	16 726 (72.3)	57 022 (69.6)	0.06	71.4	71.4	0
1	6398 (27.7)	24 849 (30.4)	0.06	28.6	28.6	0
Disclosed intimate partner violence	345 (1.5)	998 (1.2)	0.02	1.4	1.4	0
Material resources quintile, median (IQR)	3 (1-4)	3 (2-4)	0.16	2.8	2.8	0
Substance use in pregnancy						
Yes	223 (1.0)	1151 (1.4)	0.04	1.1	1.1	0
Unknown	56 (0.2)	3420 (4.2)	0.27	0.4	0.4	0
Alcohol exposure in pregnancy						
Yes	565 (2.4)	1819 (2.2)	0.02	2.50	2.5	0
Unknown	94 (0.4)	3324 (4.1)	0.25	0.5	0.5	0
Smoking status						
Patient smokes	856 (3.7)	5398 (6.6)	0.13	4.6	4.6	0
Unknown	22 (0.1)	1601 (2.0)	0.19	0.2	0.2	0
Lives with a person who smokes	2128 (9.2)	10 958 (13.4)	0.13	10.4	10.4	0
Primary language						
English	21 071 (91.1)	63 052 (77.0)	0.39	89.2	89.2	0
Unknown	1253 (5.4)	10 841 (13.2)	0.27	6.4	6.4	0
Maternal health condition						
Present	4762 (20.6)	14 745 (18.0)	0.07	20.2	20.2	0
Unknown	83 (0.4)	1370 (1.7)	0.13	0.5	0.5	0
Mental health condition						
Present	4333 (18.7)	8749 (10.7)	0.23	16.2	16.2	0
Unknown	41 (0.2)	3329 (4.1)	0.27	0.3	0.3	0
Assisted reproduction						
Any	1066 (4.6)	5501 (6.7)	0.09	5.3	5.3	0
Unknown	31 (0.1)	880 (1.1)	0.12	0.2	0.2	0
Year of pregnancy						
2012	2385 (10.3)	10 359 (12.7)	0.07	10.9	10.9	0
2013	3616 (15.6)	14 292 (17.5)	0.05	16.1	16.1	0
2014	3783 (16.4)	13 583 (16.6)	0.01	16.4	16.4	0
2015	4195 (18.1)	13 745 (16.8)	0.04	17.9	17.9	0
2016	4214 (18.2)	14 635 (17.9)	0.01	18.1	18.1	0
2017	4496 (19.4)	14 033 (17.1)	0.06	18.7	18.7	0
2018	435 (1.9)	1224 (1.5)	0.03	1.7	1.7	0

Abbreviations: BMI, body mass index (calculated as weight in kilograms divided by height in meters squared); ED, emergency department.

^a^ Weighting is by propensity-based overlap weights.

After weighting, baseline characteristics (Table 1) and most care indicators (Table 2) were balanced between the midwifery-model and the obstetrics-model groups (eFigure in Supplement 1). However, among the midwifery-model group compared with the obstetrics-model group, there was higher prenatal class attendance (14 803 [64.0%] vs 33 889 [41.4%]), higher folic acid use (21 880 [94.6%] vs 62 931 [76.9%]), and lower episiotomy rates (2 879 [12.5%] vs 15 718 [19.2%]). Type of birth also remained unbalanced, with patients in the midwifery-model group compared with patients in the obstetrics-model group experiencing higher rates of spontaneous vaginal deliveries (14 138 [61.1%] vs 44 041 [53.8%]), and lower rates of assisted vaginal deliveries (2 849 [12.3%] vs 13 902 [17%]). All women were followed up for 42 days after hospital discharge.

**Table 2.** Care Indicators According to Receipt of Midwifery-Model Care or Obstetrics-Model Care, Before and After Propensity Score Overlap Weighting

Care indicator	Before weighting			After weighting^a^		
	Patients, No. (%)		Standardized difference	Patients, %		Standardized difference
	Midwifery model (n = 23 124)	Obstetrics model (n = 81 171)		Midwifery model	Obstetrics model	
Prenatal class						
Yes	14 803 (64.0)	33 889 (41.4)	0.47	63.4	45.1	0.43
Unknown	2093 (9.1)	5811 (7.1)	0.07	9.0	6.8	0.08
Folic acid use						
Yes	21 880 (94.6)	62 931 (76.9)	0.53	94.2	83.0	0.36
Unknown	137 (0.6)	5365 (6.6)	0.33	0.64	3.1	0.18
First trimester visit						
Yes	21 166 (91.5)	69 672 (85.1)	0.20	91.7	90.0	0.06
Unknown	848 (3.7)	5362 (6.5)	0.13	3.1	3.10	0
Mode of birth						
Spontaneous vaginal	14 138 (61.1)	44 041 (53.8)	0.15	61.1	54.6	0.13
Assisted vaginal	2849 (12.3)	13 920 (17.0)	0.13	12.2	16.9	0.14
Cesarean	6137 (26.5)	23 910 (29.2)	0.06	26.7	28.50	0.04
Gestational age at birth, median (IQR), weeks	40 (39-41)	39 (38-40)	0.26	39.5	39.2	0.21
Postpartum visits provided by an OB, median (IQR), No.	0 (0-0)	0 (0-1)	0.77	0.12	0.54	0.68
Postpartum visits provided by a family physician, median (IQR), No.	0 (0-1)	1 (0-2)	0.70	0.41	1.1	0.59
Postpartum visits provided by a midwife, median (IQR), No.	7 (6-8)	NA	NA	7.0	NA	NA
Perineal laceration is 3rd or 4th degree						
Yes	1277 (5.5)	3837 (4.7)	0.04	5.4	5.0	0.02
Unknown	1573 (6.8)	6516 (8.0)	0.04	6.7	7.7	0.04
Episiotomy						
Yes	2879 (12.5)	15 718 (19.2)	0.19	12.2	17.6	0.15
Unknown	726 (3.1)	5601 (6.8)	0.17	3.1	5.5	0.12
Type of cesarean section						
Unplanned	4902 (21.2)	17 715 (21.6)	0.01	21.3	21.0	0.01
Planned	1235 (5.3)	6195 (7.6)	0.09	5.4	7.5	0.09
Unknown	16 987 (73.5)	57 961 (70.8)	0.06	73.3	71.5	0.04
Transfer of midwifery care to an obstetrician						
No	13 464 (58.2)	0	NA	NA	0	NA
Yes	9533 (41.2)	0	NA	NA	0	NA
Unknown	127 (0.5)	0	NA	NA	0	NA

Abbreviations: NA, not available; OB, obstetrician.

^a^ Weighting is by propensity-based overlap weights.

Women in midwifery-model care received a median (IQR) of 7 (6-8) postpartum visits with a midwife, compared with 0 (0-1) postpartum obstetrician visits among women receiving obstetrician-model care. Those with midwifery-model care had fewer postpartum visits by a family physician than those with obstetrician-model care (median [IQR], 0 [0-1] visits vs 1 [0-2] visits) (Table 2).

An unscheduled ED visit within 42 days postpartum was less likely in the midwifery-model group (1542 of 23 124 patients [6.7%]; aRR, 0.78; 95% CI, 0.73 to 0.83) than in the obstetrics-model group (6899 of 81 871 patients [8.4%]; ARD, 1.9%; 95% C1, −2.3% to −1.5%) (Table 3). Comparing the patients in the midwifery-model group with the patients in the obstetrics-model group, the same pattern was seen for an ED visit 7 days or less (3.2% vs 4.5%; aRR, 0.71; 95% CI, 0.65 to 0.77) and a low acuity ED visit within 42 days postpartum (1.8% vs 2.3%; aRR 0.73; 95% CI, 0.64 to 0.82), but not in risk of readmission to hospital (1.1% vs 1.1%; aRR 0.99, 95% CI, 0.85 to 1.16) (Table 3).

**Table 3.** Study Outcomes Comparing Women in Midwifery-Model Care vs Obstetrics-Model Care^a^

Outcomes	Total No.	Patients, No. (%) with outcome	Relative risk (95% CI)	Absolute risk difference, % (95% CI)
ED visit ≤42 d postpartum				
Obstetrics-model care	81 871	6899 (8.4)	1 [Reference]	1 [Reference]
Midwifery-model care	23 124	1542 (6.7)	0.78 (0.73 to 0.83)	−1.88 (−2.30 to −1.45)
ED visit ≤7 d postpartum				
Obstetrics-model care	81 871	3707 (4.5)	1 [Reference]	1 [Reference]
Midwifery-model care	23 124	745 (3.2)	0.71 (0.65 to 0.77)	−1.35 (−1.65 to −1.05)
Low acuity ED visit ≤42 d postpartum				
Obstetrics-model care	81 871	1899 (2.3)	1 [Reference]	1 [Reference]
Midwifery-model care	23 124	409 (1.8)	0.73 (0.64 to 0.82)	−0.67 (−0.89 to −0.44)
Readmission to hospital ≤42 d postpartum				
Obstetrics-model care	81 871	914 (1.1)	1 [Reference]	1 [Reference]
Midwifery-model care	23 124	247 (1.1)	0.99 (0.85 to 1.16)	−0.01 (−0.19 to 0.17)

Abbreviation: ED, emergency department.

^a^ Results are presented for the primary outcome of any ED visit between the birth hospital discharge date up to 42 days thereafter, as well as for the secondary outcomes of (1) any ED visit between the birth hospital discharge date up to 7 days thereafter, (2) any low acuity ED visit between the birth hospital discharge date up to 42 days thereafter, and (3) any hospital readmission between the birth hospital discharge date up to 42 days thereafter.

The risk of postpartum ED use differed by mode of delivery, being highest after cesarean birth (Table 4). Among women with a spontaneous vaginal delivery, those in midwifery-model care had a lower risk of an ED visit within 42 days (aRR, 0.72; 95% CI, 0.66 to 0.78), as did those undergoing assisted vaginal birth (aRR, 0.70; 95% CI, 0.60 to 0.82); no significant association was seen for cesarean birth (Table 4).

**Table 4.** Risk of an Emergency Department Visit Between the Birth Hospital Discharge Date Up to 42 Days Thereafter^a^

Outcomes	Total No.	Patients with outcome, No. (%)	aRR (95% CI)	Adjusted absolute risk difference, % (95% CI)
Spontaneous vaginal delivery				
Obstetrics-model care	44 041	2923 (6.6)	1 [Reference]	1 [Reference]
Midwifery-model care	14 138	691 (4.9)	0.72 (0.66 to 0.78)	−1.91 (−2.40 to −1.43)
Assisted vaginal delivery				
Obstetrics-model care	13 920	1308 (9.4)	1 [Reference]	1 [Reference]
Midwifery-model care	2849	192 (6.7)	0.70 (0.60 to 0.82)	−2.95 (−4.12 to −1.77)
Cesarean delivery				
Obstetrics-model care	23 910	2668 (11.2)	1 [Reference]	1 [Reference]
Midwifery-model care	6137	659 (10.7)	0.95 (0.87 to 1.04)	−0.48 (−1.42 to 0.46)
Intrapartum transfer of care from a midwife to an obstetrician				
No transfer of care				
Obstetrics-model care	81 871	6899 (8.4)	1 [Reference]	1 [Reference]
Midwifery-model care	13 591	751 (5.5)	0.65 (0.60 to 0.70)	−2.87 (−3.35 to −2.39)
Transfer of care				
Obstetrics-model care (not applicable)	NA	6899 (8.4)	1 [Reference]	1 [Reference]
Midwifery-model care	9533	791 (8.3)	0.97 (0.90 to 1.05)	−0.39 (−0.98 to 0.19)

Abbreviations: aRR, adjusted relative risk; NA, not applicable.

^a^ Compared women in midwifery-model care vs obstetrics-model care, further stratified by mode of delivery, as well as intrapartum transfer of pregnancy care from a midwife to an obstetrician.

Among the 23 124 women in the midwifery-model group, 9533 (41%) had a transfer of care intrapartum to an obstetrician (Table 4). A lower relative risk of ED use was seen comparing midwifery-model vs obstetrics-model among women who did not require transfer of care, but not among those who did (Table 4).

## Discussion

In this study, approximately 1 in 15 women receiving midwifery-model care and 1 in 12 women receiving obstetrician-model care visited the ED postpartum among women who were low-risk, primiparous, and gave birth in an Ontario hospital. A lower risk of ED use postpartum was most pronounced among women who had midwifery-model care, especially among those with a spontaneous or assisted vaginal birth and those who maintained midwifery care intrapartum. For cesarean births, or following intrapartum transfer of care, these significant differences no longer persisted.

The current findings were supported by the findings of other studies,^1,13,19,20^ which suggested various benefits from postpartum midwife visits, including higher rates of breastfeeding, patient satisfaction, and uptake of medical and educational interventions. While there may be multiple reasons for less postpartum ED use among patients receiving midwifery care, explanations include the intensive postpartum visit schedule and 24-hour on-call access afforded by a midwife, as demonstrated herein by a higher median number of postpartum visits in the midwifery-model vs obstetrics-model groups. Some factors of postpartum ED use may be associated with birth location (eg, rural vs urban residence), mode of birth, and immigration status.^6^

While care indicators were largely similar between the 2 groups after overlap weighting, some differences persisted, including gestational age at birth, receipt of an episiotomy and mode of birth, each of which may reflect midwifery practices. These differences reflected the fact that midwives tend to tolerate induction of labor at a later gestational age, do not offer routine episiotomy, and promote lower rates of medical intervention. Higher rates of prenatal class attendance and folic acid use among patients receiving midwifery care may also reflect a more engaged patient population.^21^

### Limitations

This study has limitations, including the observational study design. While we tried to reduce confounding by indication by using a propensity-based weighting approach, causality cannot be inferred. The reason for an ED visit was not evaluated; however, another Ontario study highlighted abdominal pain (22%), wound-related issues (13%), and urinary issues (10%) as the main reasons for ED use soon after birth,^22^ similar to findings from Alberta, Canada.^23^ Variation in episiotomy use and mode of birth that persisted after matching may drive some of the observed differences in postpartum ED visits. However, this may also reflect an unmet need after the birth hospital discharge for the provision of routine wound care, something that midwives provide during their postpartum visits. In addition, differences in ED visits also persisted for low acuity ED visits, which are not expected to include serious medical conditions directly associated with mode of delivery or episiotomy care. As our study data were available only up to 2018, the influence of the COVID-19 pandemic on practice tendencies was not ascertainable. To create homogeneous and comparable exposure groups, only primiparous women were included. This study also excluded women who received family physician-model care, whose practice patterns vary considerably, and which may differ from that provided by midwives or obstetricians.^24^ Between 10% to 20% of Ontarian women in midwifery-model care give birth outside a hospital setting, and represent a particularly low-risk population.^25,26^ These women were excluded since obstetricians do not perform out-of-hospital deliveries. Finally, we required a woman to have a recorded BMI, since a high BMI is an important factor of adverse obstetric outcomes.^27^ Together, such exclusions may limit the generalizability of the current study findings to other groups of women and their clinical practitioners. Certainly, more research is needed to evaluate whether the current findings are similar to family-physician model care, or among parous women.

## Conclusions

In this cohort study of 104 995 women who were low risk and primiparous, midwifery-model care was associated with less postpartum ED use than a conventional obstetrics-model care model. One goal of Ontario’s Midwifery Program is to provide high-quality postpartum care.^1^ While all women in Ontario have access to fully funded antenatal, intrapartum, and postpartum care, the emphasis on early postpartum care under the provincially funded and regulated midwifery model may be associated with a reduction in ED use postpartum. Consideration of improving access to postpartum care for patients receiving obstetrics-model care could address an unmet need for some patients. This study may also inform maternal-newborn health policy across Canada, particularly in terms of discussions associated with the benefits of expanding a midwifery or nurse-based interdisciplinary postpartum care model, to meet the needs of new mothers.

## Notes

Supplement 1. **eFigure.** Density Plots for the Propensity Scores for Receipt of Midwifery-Model Care and Obstetrics-Model Care, Before and After Overlap Weighting

Supplement 2.
Data Sharing Statement
