# Supplementary material for: Postpartum Emergency Department Use Following Midwifery-Model vs Obstetrics-Model Care
Source: JAMA Netw Open. 2024 Apr 29;7(4):e248676. doi: 10.1001/jamanetworkopen.2024.8676 (PMC11059030; doi:10.1001/jamanetworkopen.2024.8676)
Supplement: Supplement 1. — eFigure. Density Plots for the Propensity Scores for Receipt of Midwifery-Model Care and Obstetrics-Model Care, Before and After Overlap Weighting [file jamanetwopen-e248676-s001.pdf]

Supplemental Online Content

Sorbara C, Ray JG, Darling EK, Chung H, Podolsky S, Stukel TA. Postpartum emergency department use following midwifery-model vs obstetrics-model care. *JAMA Netw Open*. 2024;7(4):e248676. doi:10.1001/jamanetworkopen.2024.8676

eFigure 1. Density Plots for the Propensity Scores for Receipt of Midwifery-Model Care and Obstetrics-Model Care, Before and After Overlap Weighting

This supplemental material has been provided by the authors to give readers additional information about their work.

© 2024 Sorbara C et al. *JAMA Network Open*.

eFigure 1. Density plots for the propensity scores for receipt of midwifery-model care (red line) and obstetrics-model care (blue line), before and after overlap weighting.

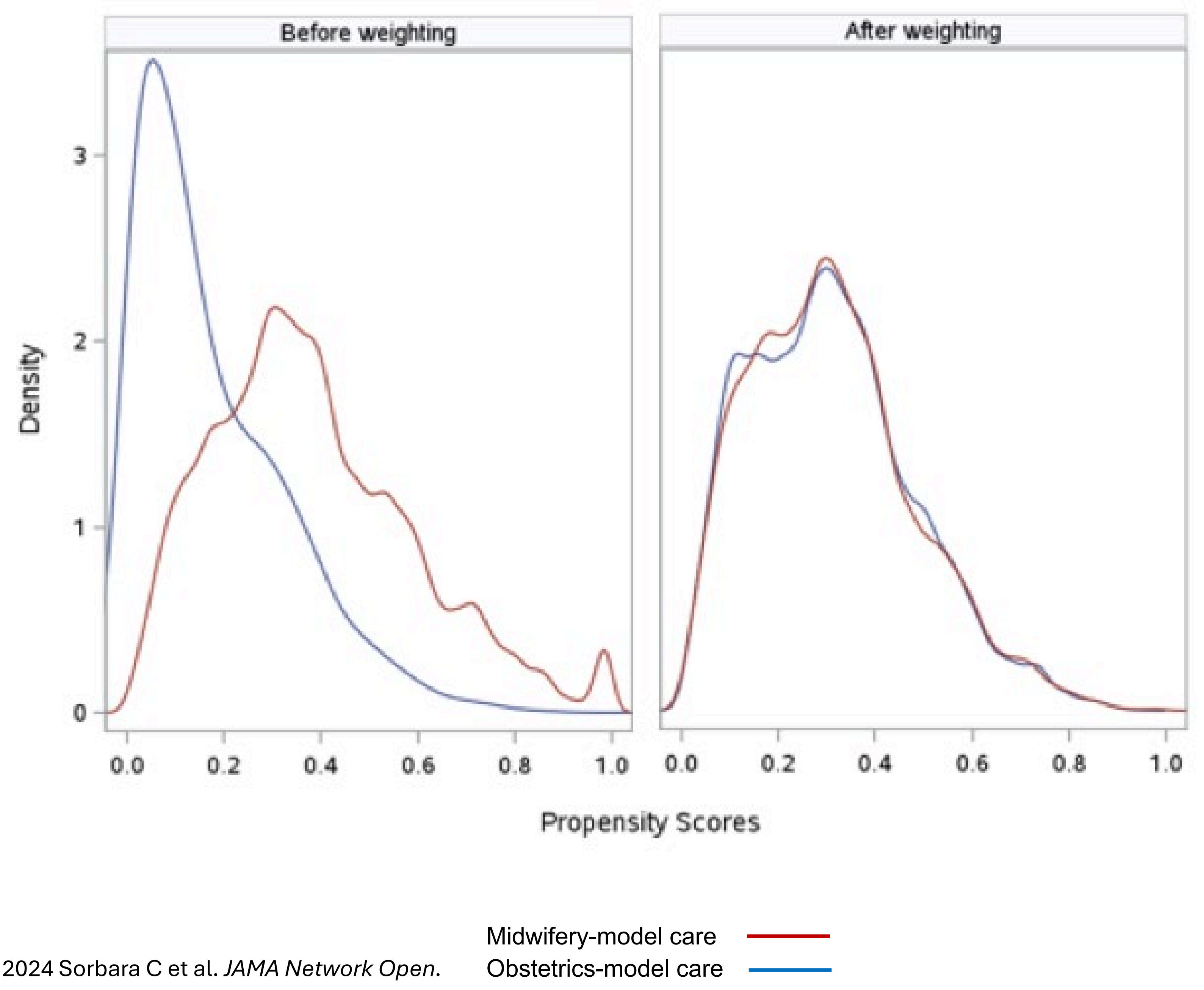

© 2024 Sorbara C et al. *JAMA Network Open*.
